# Genetic diversity of Caracu cattle reveals contrasting trends between general and closed populations

**DOI:** 10.1007/s11250-026-05351-9

**Published:** 2026-10-08

**Authors:** Gustavo Henrique Carvalho Borges, Mário Luiz Santana, Viviane Andrade Ligori, Mayne Barboza Sarti, Eula Regina Carrara, Júlia de Paula Soares Valente, Maria Eugênia Zerlotti Mercadante, Lenira El Faro Zadra

**Affiliations:** 1 School of Agricultural and Veterinary Sciences, São Paulo State University (UNESP), Jaboticabal, SP Brazil; 2 Animal Science Institute, Beef Cattle Research Center, Sertãozinho, SP Brazil; 3 Federal University of Rondonópolis, Rondonópolis, MT Brazil

**Keywords:** *Bos taurus*, Conservation genetics, Kinship networks, Locally adapted breeds, Small populations

## Abstract

The conservation of genetic diversity and control of inbreeding rate are fundamental factors in animal breeding programs, especially programs for adapted cattle breeds such as Caracu. However, there are still knowledge gaps regarding the population structure of this breed as a whole and the genetic dynamics in subpopulations, like closed populations reared in experimental farms in Brazil. Within this context, this study aimed to evaluate the population structure of the Caracu cattle breed by investigating inbreeding levels and genetic diversity based on pedigree analyses. Two populations were analyzed: a general population (183,326 animals; 1900–2023) and a closed subpopulation selected exclusively for body weight for more than four decades and maintained by the Institute of Animal Science, Sertãozinho, São Paulo, Brazil (4,892 animals; 1977–2023). Population structure and pedigree depth were evaluated through the estimation of several parameters, like generation intervals, equivalent complete generations, pedigree depth, inbreeding levels, and diversity loss. The mean inbreeding coefficient of the general population was higher (2.6%) than that of the subpopulation (2.35%), with maximum values of 44.3% and 25%, respectively. The subpopulation exhibited greater pedigree completeness, higher average coancestry, and lower genetic diversity, indicating cumulative losses due to genetic bottlenecks and unequal founder contributions. In the general population, kinship network analysis revealed the presence of highly connected individuals, suggesting intensive use of certain sires and the formation of distinct family clusters. In contrast, a more uniform kinship structure was observed in the subpopulation. The results provide important support for the design of mating strategies and the improvement of conservation and breeding programs for the Caracu breed.

**Supplementary Information:**

The online version contains supplementary material available at https://doi.org/10.1007/s11250-026-05351-9.

## Introduction

The conservation of animal genetic resources is a global concern because of the increasing losses of diversity observed in various breeds and local populations. Conservation programs have been developed in an attempt to prevent extinction and to preserve genetic diversity; the latter is typically quantified using parameters such as inbreeding, average relatedness, effective population size, and probabilities of gene origin. According to the Food and Agriculture Organization of the United Nations (FAO 2021), approximately 174 cattle breeds have become extinct, while about 22% of the breeds known in 2021 were at risk of extinction. The risk status of about 48.9% of breeds remains unknown, mainly because of the lack of reliable data on their population structure and genetic diversity (Pezzini et al. 2018; FAO 2021).

The evaluation of population structure and the control of inbreeding rate are leading challenges in cattle breeding programs. Although fundamental for genetic progress, intensive selection may compromise genetic diversity within a breed because it can increase homozygosity, reduce the effective population size, promote genetic bottlenecks, and increase the probability of expression of deleterious alleles due to higher homozygosity (Rebelato and Caetano 2018; Bosse et al. 2019). In the long term, such effects may negatively impact the adaptability, reproductive health, and productive performance of animals and are of particular concern in the case of local breeds or small populations. The management of genetic diversity should therefore be a priority in any genetic selection strategy, especially for breeds under conservation or with a history of population restriction (Baes et al. 2019).

The loss of genetic diversity and the increase in homozygosity caused by inbreeding are important concerns in local breeds or small populations, which are more susceptible due to their reduced effective population size, restricted gene pool, and greater probability of inbreeding. The Caracu breed (*Bos taurus*) is a cattle breed that was formed by different crossings of animals introduced by European colonizers in Brazil in the 16th and 17th centuries. Brazilian autochthonous breeds emerged in diverse tropical and subtropical environments and are commonly described as locally adapted breeds of Iberian origin (Verardo et al. 2021). Historically, the Caracu breed has been extensively used in Brazilian livestock production because of its rusticity, docility, and versatility. These animals are widely used for draft work and milk and beef production and as a base for crossbreeding. The breed came to prominence in the 1940s and 1950s, when it became one of the most important breeds in Brazilian cattle production (Trovo and Duarte 1981; Mercadante 2005).

Despite its zootechnical relevance, there has been a sharp decline in the Caracu breed population starting in the 1960s, which was associated with the closure of the herd book and the introduction of Zebu cattle in Brazil. In addition, the combined effects of directional selection, reduction in the number of breeding individuals, and unequal parental contributions may have increased inbreeding in the herds. This increase in homozygosity and loss of genetic diversity contributed to inbreeding depression in reproductive traits and to the reduction in the number of animals of the breed (Lima et al. 1992; Pereira et al. 2005; Abduch et al. 2022).

In response to this critical scenario, the Institute of Animal Science (IZ) of the State of São Paulo implemented a genetic improvement program for the Caracu breed in 1976, which has focused on the preservation of genetic material and the improvement of productive performance, specifically weight at yearling age, thereby contributing to restructuring of the base population (Pereira et al. 2005). Since then, bulls are officially evaluated for weight at 378 days of age under feedlot conditions and cows are evaluated for weight at 550 days of age under pasture conditions (Bonilha et al. 2008; Ligori et al. 2025). Other phenotypes are also routinely collected for research and future evaluations, including carcass traits, feed efficiency, weights at different ages, and reproductive traits (Abduch et al. 2022, 2024; Rodrigues et al. 2024; Ligori et al. 2025), making IZ a benchmark in the genetic evaluation and maintenance of the breed to this day.

The number of Caracu herds has increased in Brazil in the 1980s, with approximately 12,386 animals distributed across 28 herds (Trovo and Duarte 1981). Currently, publicly available information indicates that approximately 30,000 animals were actively registered in the breed population at the time of the assessment (Abduch et al. 2022). Despite these advances, challenges related to inbreeding in the Caracu breed still persist, especially because of the limited availability of sires in artificial insemination centers according to information from the Brazilian Association of Caracu Breeders (ABC Caracu 2022). To avoid bottlenecks in the breed, management strategies must be implemented to ensure the maintenance of genetic diversity without compromising the gains achieved in productive performance.

Considering the historical importance of the Caracu breed in tropical countries and the limited information on its population structure, this study aimed to evaluate the population structure of the breed based on pedigree data, analyzing the general population (i.e., the total population registered with ABC Caracu), and a closed subpopulation maintained by IZ in Brazil. Additionally, this study characterized the inbreeding dynamics and other population parameters over time in order to support mating and selection strategies, as well as strategies for the conservation of genetic diversity in the breed.

## Materials and methods

### Animals and data collection

The pedigree file of the general population of Caracu cattle is maintained by the Embrapa Beef Cattle Genetic Improvement Program - Geneplus (Geneplus-Embrapa Program) and was provided by ABC Caracu for this study. The file contained 183,326 animals born between 1900 and 2023, including 3,802 sires and 55,757 dams. Additionally, the pedigree file trimmed for the subpopulation of Caracu cattle from IZ was analyzed. This file contained 4,892 animals born between 1977 and 2023, including 278 sires and 1,347 dams.

The herds used for the general population were distributed across the Brazilian states of Acre, Amazonas, Bahia, Ceará, Federal District, Espírito Santo, Goiás, Maranhão, Minas Gerais, Mato Grosso do Sul, Mato Grosso, Pará, Pernambuco, Paraná, Rio de Janeiro, Rondônia, Rio Grande do Sul, Santa Catarina, São Paulo, and Tocantins. The general population also comprised herds located in Paraguay. The selection of animals mainly focused on beef production, although few herds were selected for milk production or dual-purpose according to the selection objectives of each farmer.

The Caracu subpopulation is maintained by IZ on an experimental farm in the municipality of Sertãozinho, São Paulo state, and its breeding program was designed for beef production. Currently (2026), the IZ subpopulation consists of 180 cows and 8 bulls. Half of the bulls are 2 years old (1st breeding season), and the other half are 3 years old (2nd breeding season). The culling rate is 20% per year for females and 50% for males. Therefore, while this subpopulation is predominantly managed as a closed herd, specific management decisions led to the introduction of external genetics; for instance, 31 polled animals (2 males and 29 females) were introduced into the herd between 2016 and 2018 to incorporate the polled phenotype.

Both the general population and the IZ subpopulation have genetic evaluation programs, in which breeding values are estimated using the best lienar unbiased prediction (BLUP) methodology and used to support selection decisions (Aguilar et al. 2010; Christensen and Lund 2010).

### Pedigree analysis

The pedigree information consisted of identification of the animal and its parents, year of birth, and sex. The consistency of the pedigree file was checked using the CFC software (Sargolzaei et al. 2006) to identify and correct possible errors such as duplicate and bisexual animals and looping.

The generation interval (* GI*), defined as the mean age of the parents at the birth of offspring kept for reproduction, was calculated using the RelaX2 software (Strandén 2014). The * GI* was calculated along the four gametic pathways: sire to son ($\:{l}_{ss}$), sire to daughter ($\:{l}_{sd}$), dam to son ($\:{l}_{ds}$), and dam to daughter ($\:{l}_{dd}$). The four * GI* were based on the recorded birth dates of the animals, along with those of their sires and dams. The mean * GI* ($\:\stackrel{-}{l}$) was calculated as follows:

$$\:\stackrel{-}{l}=\frac{{l}_{ss}+{l}_{sd}+{l}_{ds}+{l}_{dd}}{4}.$$

Based on the calculated mean * GI*, the reference population was defined as animals born within the most recent period equivalent to one * GI* to ensure adequate pedigree depth (6.16 for the general population and 5.12 for the subpopulation). Using animals from the most recent * GI* ensures that the reference population has adequate pedigree depth and reflects current genetic trends, avoiding bias from older animals with incomplete records (Eynard et al. 2018).

The remaining metrics are described below and were estimated using the RelaX2 (Strandén 2014) and PEDIG (Boichard 2002) software. For both populations, the respective reference population was used for metric estimation.

#### Equivalent complete generations

The number of equivalent complete generations (* ECG*) for individual * i* $\:\left(EC{G}_{i}\right)$ was defined as the sum over all distinct known ancestors:

$$\:{ECG}_{i}={\sum\:}_{j\in\:{A}_{i}}{\left(\raisebox{1ex}{$1$}\!\left/\:\!\raisebox{-1ex}{$2$}\right.\right)}^{{g}_{ij}},$$

where $\:{A}_{i}$ represents the set of unique ancestors in the pedigree of animal * i*, and $\:{g}_{ij}$ is the number of generations separating * i* from ancestor * j* (Maignel et al. 1996). This metric quantifies the depth and completeness of pedigree information by weighting each ancestor according to its genetic distance, with parents ($\:{g}_{ij}=1$) contributing 1/2, grandparents ($\:{g}_{ij}=2$) contributing 1/4, and so on, and provides a continuous measure of the amount of ancestral information available.

#### Inbreeding, coancestry, and degree of non-random mating parameters

The mean inbreeding coefficient (* F*) was calculated using a modified version of Wright’s coefficient (1969), as proposed by Meuwissen and Luo (1992). Individual inbreeding increments ($\:\varDelta\:{F}_{i}$) were calculated following Falconer and Mackay (1996), with modifications proposed by González-Recio et al. (2007) and Gutiérrez et al. (2009):

$$\:{\varDelta\:F}_{i}=1\:-\sqrt[t-1]{1-{F}_{i}},$$

where $\:{F}_{i}$ is the inbreeding coefficient for animal *i*, and * t* is the equivalent number of complete generations for this individual (Maignel et al. 1996; Boichard et al. 1997). The expression relating inbreeding in generation *t* ($\:{F}_{t}$) to the inbreeding rate ($\:\varDelta\:F$) proposed by Gutiérrez et al. (2009) is:

$$\:{F}_{t}=1-(1-\varDelta\:F{)}^{t-1}.$$

Inbreeding can also be related to the average coancestry (* C*) within a population since populations with increased coancestry tend to have a higher probability of mating between related individuals. Thus, an increase in average coancestry is directly associated with the expected increase in inbreeding in subsequent generations, even under random mating, while non-random mating schemes can enhance this effect (Cervantes et al. 2011).

The degree of non-random mating ($\:\alpha\:$) was estimated as the correlation of genes within individuals in relation to the correlation of randomly selected genes from the population, as proposed by Caballero and Toro (2000). This coefficient indicates the degree of deviation from Hardy-Weinberg proportion and is related to * F*. Coancestry was defined as the probability that two alleles randomly sampled from two individuals are identical by descent and was calculated as proposed by Wright (1969) and modified by Cervantes et al. (2011):

$$\:1-F=(1-C)(1-\alpha\:),$$

where $\:C\:$is the average coancestry coefficient among all individuals, which is calculated in pairs (Cervantes et al. 2011) and represented by:

$$\:{C}_{ba}=1-\sqrt[\frac{tb+ta}{2}]{1-{C}_{ba}},$$

where * tb* and * ta* are the number of * ECG* in individuals * a* and * b*, respectively, and $\:{C}_{ba}$ is the average coancestry between individuals * b* and * a*. In addition, the $\:F\:$value for the animals was divided into two classes, called old * F* ($\:{F}_{old}$) and recent $\:F\:\left({F}_{new}\right)$. $\:{F}_{new}$ was defined as the period of the three generations closest to the evaluated individuals, and $\:{F}_{old}$ was calculated as follows:

$$\:{F}_{old}=\:{F}_{total}-{F}_{new}.$$

#### Effective population size

The effective population size ($\:{N}_{e}{F}_{i}$) was estimated as proposed by Gutiérrez et al. (2009) based on the individual increase in the inbreeding coefficient ($\overline {\Delta F}$):

$$\:{N}_{e}{F}_{i}=\frac{1}{\stackrel{-}{2\varDelta\:F}},$$

where $\:\stackrel{-}{\varDelta\:F}$ is the average $\:\varDelta\:Fi$.

#### Effective number of founders, ancestors, and founder genomes and loss of genetic diversity

The number of founders ($\:{f}_{e}$) and ancestors ($\:{f}_{a}$) was obtained to assess the concentration of the origin of both populations (general and subpopulation). Parameter $\:{f}_{e}$ was defined as the number of founders that contribute equally and would be expected to produce the same genetic diversity observed in the study population (Lacy 1989). This parameter was calculated as:

$$\:{f}_{e}=\frac{1}{\sum\:_{i=1}^{f}{q}_{k}^{2}},$$

where $\:{q}_{k}\:$is the probability of genetic descent from founder * k*.

Parameter $\:{f}_{a}$ is the minimum number of ancestors required to account for the genetic diversity of a population and was calculated as:

$$\:{f}_{a}=\frac{1}{\sum\:_{j=1}^{a}{q}_{j}^{2}},$$

where $\:{q}_{j}$ is the marginal contribution of ancestor * j*, which is the genetic contribution made by an ancestor that is not explained by other previously selected ancestors.

The effective number of founder genomes ($\:{f}_{g}$) can be defined as the number of founders that would be expected to produce the same genetic diversity observed in the population studied if the founders were equally represented and there was no loss of alleles (Ballou and Lacy 1995). Following Caballero and Toro (2000), parameter $\:{f}_{g}$ was obtained as the inverse of twice the average kinship of individuals included in a predefined reference population. Parameter $\:{f}_{a}$ complements the information provided by $\:{f}_{g}$ by accounting for losses in genetic diversity caused by the unequal use of breeding individuals, which creates genetic bottlenecks. In addition, the more equally founders contribute to the reference population, the higher is $\:{f}_{g}$.

The effective number of non-founders ($\:{f}_{n}$) accounts for the effects of genetic drift in non-founder generations. This parameter was computed as a function of $\:{f}_{e}$ and $\:{f}_{n}$ (Caballero and Toro 2000):

$$\:\frac{1}{{f}_{g}}=\:\frac{1}{{f}_{e}}+\:\frac{1}{{f}_{n}}.$$

Genetic diversity (* GD*) in the reference population was calculated as follows (Lacy 1989; 1995):

$$\:GD=1-\frac{1}{{f}_{g}}.$$

In domesticated populations like cattle, genetic bottlenecks and genetic drift occur frequently and lead to the loss of genetic diversity. The loss of genetic diversity in the population since the founding generation is estimated as * 1-GD*. The loss of genetic diversity due to unequal contributions from founders is estimated as $\:1-{GD}^{*}$ (Caballero and Toro 2000):

$$\:{GD}^{*}=1-\:\frac{1}{fe},$$

where $\:{GD}^{*}$ is genetic diversity due to unequal contributions from founders.

The difference between * GD* and $\:{GD}^{*}\:$indicates the loss of genetic diversity due to genetic drift that has accumulated since the establishment of the population (Lacy 1995). This difference can be expressed as:

$$\:{GD}^{*}-\:GD=\:\frac{1}{2{f}_{n}}.$$

The contributions of common ancestors to inbreeding, i.e., those that contribute to inbreeding pathways, were calculated as described by Colleau and Sargolzaei (2008). The expected marginal contribution of each main ancestor *j* was calculated as its expected genetic contribution, independent of the contributions of other ancestors (Boichard et al. 1997).

#### Kinship network

The kinship network was based on the numerator relationship matrix A (Henderson 1976), which was constructed separately for the general population and the subpopulation. A subset of animals accounting for at least 50% of the genetic diversity were selected from each population and are represented as nodes in the undirected graph network. First, the kinship between the key ancestors and all individuals in the population was extracted from matrix A. A connection (edge) between a key ancestor and another individual was established if the kinship coefficient was ≥ 0.0625. This value corresponds to a closer kinship such as first cousins or great-great-grandparents. Lower kinship coefficients tend to have less practical impact on the management of genetic diversity (Gautason et al. 2023) and contribute to an excessively dense network when represented graphically. Thus, this threshold permits to balance the biological relevance of kinship ties and the clarity of visualization.

The internal structure of the network was investigated using a community detection algorithm. The Louvain method (Blondel et al. 2008) was used for this purpose, which identifies groups of nodes (in this case, families) that are more strongly connected to each other than to the rest of the network. Thus, the method permits to identify subsets of individuals that are more strongly related within the population. Within this context, modularity (* Q*) is a scalar value ranging from − 1 to 1 that measures the density of connections within communities compared to connections between communities. For weighted networks, *Q* is calculated using the following equation (Albert and Barabási 2002):

$$\:Q=\:\frac{1}{2m}\:{{\Sigma\:}}_{ij}[{A}_{ij}-\frac{{k}_{i}{k}_{j}}{2m}]\delta\:\left({c}_{i}{c}_{j}\right),$$

where $\:m\:$represents the sum of the weights of all edges in the network; $\:{A}_{ij}$ is the weight of the edge between nodes * i* and * j*; $\:{k}_{i}$ and $\:{k}_{j}$ are the sums of the weights of the edges connected to nodes * i* and * j*; $\:{c}_{i}$ and $\:{c}_{j}$ are the communities to which nodes * i* and * j* belong, and $\:\delta\:\left({c}_{i}{c}_{j}\right)\:$is the Kronecker delta function.

The Louvain algorithm is based on a hierarchical approach. First, each node is assigned to an independent community. At each step, nodes are reassigned to communities in a local and precise manner: each node is moved to the community with which it achieves the highest contribution to modularity. The change in modularity ($\:{\Delta\:}Q$) when moving node * i* to community *C* is efficiently calculated as follows (Barbási, Watts & Newman, 2006):

$$\:{\Delta\:}Q=[\frac{{{\Sigma\:}}_{in}+{2k}_{i,in}}{2m}-(\frac{{{\Sigma\:}}_{tot}+{k}_{i}}{2m}{)}^{2}]\:-[\frac{{{\Sigma\:}}_{in}}{2m}-(\frac{{{\Sigma\:}}_{tot}}{2m}{)}^{2}-(\frac{{k}_{i}}{2m}{)}^{2}],$$

where $\:{\varSigma\:}_{in}$ is the sum of the weights of the edges within * C*; $\:{{\Sigma\:}}_{tot}$ is the sum of the weights of the edges incident to the nodes in * C*; $\:{k}_{i}$ is the sum of the weights of the edges incident to node * i*, and $\:{k}_{i,\:in}$ is the sum of the weights of the edges from node * i* to the nodes in * C*. The process stops when no node can be reallocated in such a way that would increase modularity. Subsequently, each community is aggregated into a single node, and the process is repeated with the new network, seeking hierarchical optimization. These communities were interpreted as representing families or genetic lineages structured around key ancestors.

Network analysis was conducted using *igraph* (Blondel et al. 2008) and the network topology was visualized using *ggraph*, both packages of the R software (R Core Team 2026). For spatial arrangement of the nodes, the Fruchterman-Reingold force-directed algorithm was used, which positions the nodes to minimize edge crossings and visually cluster the most connected nodes.

## Results

The genetic structure and inbreeding parameters of the Caracu breed were evaluated based on the pedigree analysis of a general population and a subpopulation selected for body weight. The reference population, defined as animals born in the last * GI*, consisted of 33,391 animals in the general population and 1,239 in the subpopulation. The maximum average pedigree depth in the general population, expressed as * ECG*, was 7.39 in 2023. In the subpopulation, the maximum value was 8.40 also in 2023. Although the subpopulation exhibited larger numbers of * ECG*, indicating greater pedigree completeness, the values were similar between the populations evaluated. The maximum number of generations was 22 and 17 for the general population and subpopulation, respectively, indicating differences in pedigree structure between populations.

In the general pedigree, the first recorded birth dated back to the early 20th century; however, it was only from the 1940s onward that there was a higher frequency of individuals (69 in 1944; 70 in 1950). Annual births increased considerably in the 1970s, exceeding 5,000 in 1992, with a peak of 9,087 in 2002. After this peak, there was a gradual decline, with approximately 4,000 annual births from 2010 to 2015 and a more pronounced drop in recent years, reaching 2,200 in 2023. In the subpopulation, the number of recorded births oscillated over the period analyzed. Between 1977 and the early 1980s, the number of births per year was low (3 in 1977; 27 in 1978; 53 in 1979) compared to the general population. There was a consistent increase from 1990, exceeding 100 births per year, with peaks being reached in 1996 (129), 1999 (133), and 2005 (149).

The $\:F\:$value was higher in the general population, where 24,646 of the 33,391 animals in the reference population (approximately 74%) were inbred, with a maximum * F* value of 44.33%. In the subpopulation, 867 of the 1,239 animals in the reference population (approximately 70%) exhibited some level of * F*, with a maximum value of 25%. When only inbred individuals were considered, the mean * F* values were similar in the two groups (2.60 and 2.35% for general and subpopulation). The distribution of individuals by inbreeding class showed that most individuals belonged to the inbreeding classes with the lowest values in both the general population (55.57%) and the subpopulation (62.9%). The percentage of highly inbred individuals was 3.66% in the general population but only 0.88% in the subpopulation.

The subpopulation showed higher total genetic diversity loss $\:(1\:-\:GD)$, which was primarily driven by increased genetic drift $\:(GD*\:-\:GD)$. There was a smaller contribution of unequal founder representation $\:(1\:-\:GD*)$, although to a lesser extent compared to the other measures of diversity loss analyzed. Table 1 shows the complete description of these metrics for the populations analyzed.

**Table 1:** Population structure metrics for the reference populations: general population and subpopulation of Caracu cattle

Metrics*	General population	Subpopulation
Number of animals in general population	183,326	4,892
Generation interval (general population)	6.16	5.05
Number of animals in reference population	33,391	1,239
Number of inbred animals	24,646	867
* ECG*	5.94	7.54
Maximum number of generations tracked	22.00	17.00
F (%)	2.60	2.35
ΔFi (%)	0.51	0.52
Mean F of inbred animals (%)	3.52	3.36
GD* – GD (%)	0.81	4.07
1 – GD* (%)	0.34	0.85
1 – GD (%)	1.15	4.92
F = 0 (% of animals)	26.19	30.00
0 < F ≤ 5 (% of animals)	55.57	62.90
5 < F ≤ 10 (% of animals)	14.58	6.21
F > 10 (% of animals)	3.66	0.88
Maximum F (%)	44.34	25.00

*ECG = equivalent complete generations; F = average inbreeding coefficient; ΔFi = average increase in inbreeding; GD* – GD = loss of genetic diversity due to genetic drift accumulated over non-founder generations; 1 – GD* = loss of genetic diversity due to unequal contributions of founders; 1 – GD = loss of genetic diversity since the founder generation

The results for * C*,$\:\:\alpha\:$, and the parameters related to genetic diversity loss revealed differences between the general population and subpopulation and are presented in Table 2. The subpopulation exhibited higher values of * C* and greater losses of genetic diversity, with reductions in * fe*, * fa*, and * fg*.

The $\:\alpha\:$ values indicate distinct mating patterns among the populations. The $\:{f}_{a}/{f}_{e}$ and $\:{f}_{g}/{f}_{e}$ ratios, as well as the cumulative number of ancestors required to explain 25%, 50%, 75%, and 95% of the current genetic diversity, were lower in the subpopulation.

**Table 2:** Coancestry, mating, genetic diversity, and ancestral contributions for the reference populations: general population and subpopulation

Metrics*	General population	Subpopulation
$\:C\:\left(\%\right)$	1.2	4.9
$\:\alpha\:$	0.0132	-0.0268
* NeFi*	92.90	86.68
* Ne*	2,907	197
$\:{f}_{e}$	146.55	58.83
$\:{f}_{a}$	106.22	31.87
$\:{f}_{g}$	43.60	10.17
$\:{f}_{a}/{f}_{e}$	0.73	0.54
$\:{f}_{g}/{f}_{e}$	0.30	0.17
A25%	13	4
A50%	46	11
A75%	159	23
A95%	1000	58

* * C* = average coancestry; $\:\alpha\:$ = degree of non-random mating; * NeFi* = effective population size based on individual increase in inbreeding; * Ne* = number of founders; * fe* = effective number of founders; * fa*= effective number of ancestors; * fg* = effective number of founder genomes; A25%, A50%, A75%, A95% = numbers of ancestors explaining a given proportion of the population’s genetic diversity

**Fig. 1:**
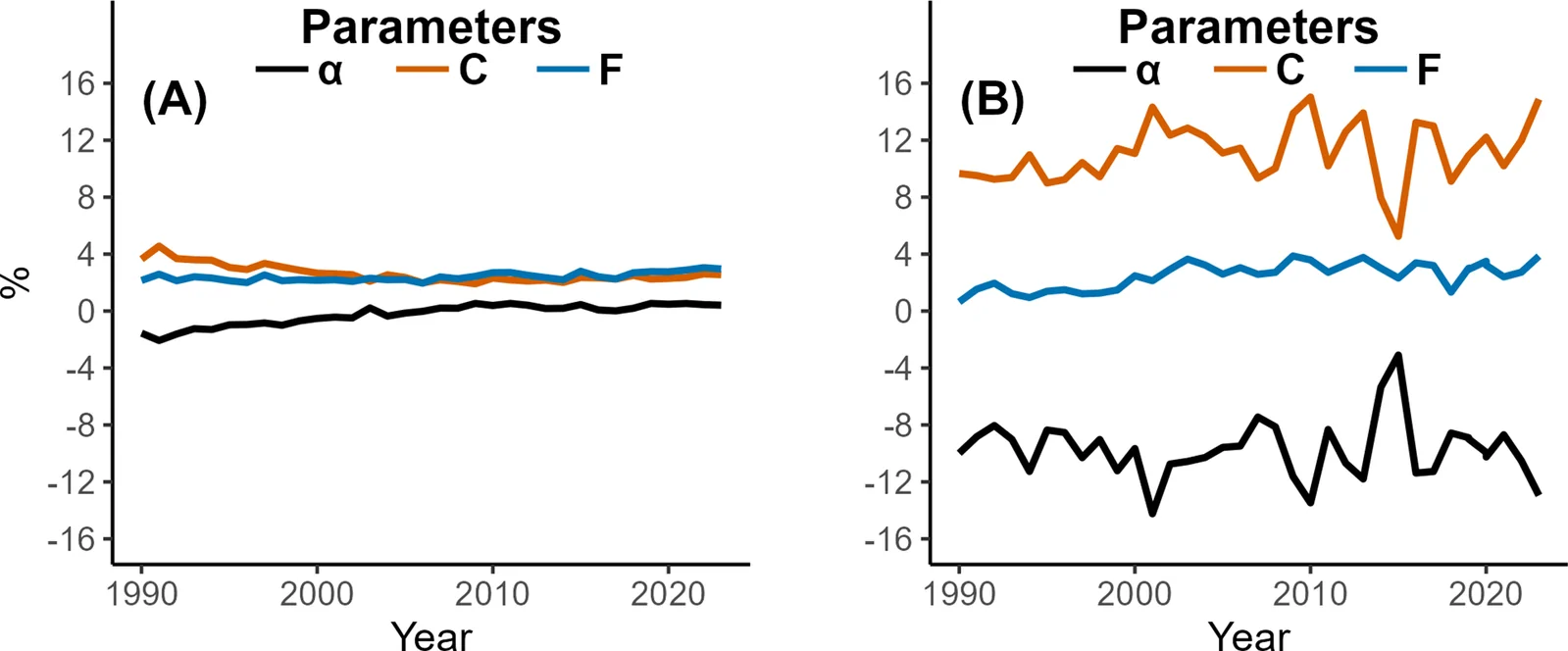
Average inbreeding coefficient (F), average coancestry coefficient (C), and degree of non-random mating (α), all in percentage (%), by birth year from 1990 to 2023 in the reference populations of the Caracu breed: (**A**) general population and (**B**) subpopulation

The annual trends in * F*, * C*, and $\:\alpha\:$ in the general population and in the subpopulation of the Caracu breed between 1990 and 2023 (Fig. 1) indicate consistent differences between these populations in terms of both mean levels and variation over the years. The subpopulation exhibited the highest * C* and negative values of $\:\alpha\:$, whereas * F* levels remained relatively stable in both populations. This trend was accompanied by an irregular increase in * C*, which measures the probability that two alleles from different individuals are identical by descent, with a peak value of 15.03% in 2010. Values of *α* remained negative throughout the period, with oscillations over the years.

**Fig. 2:**
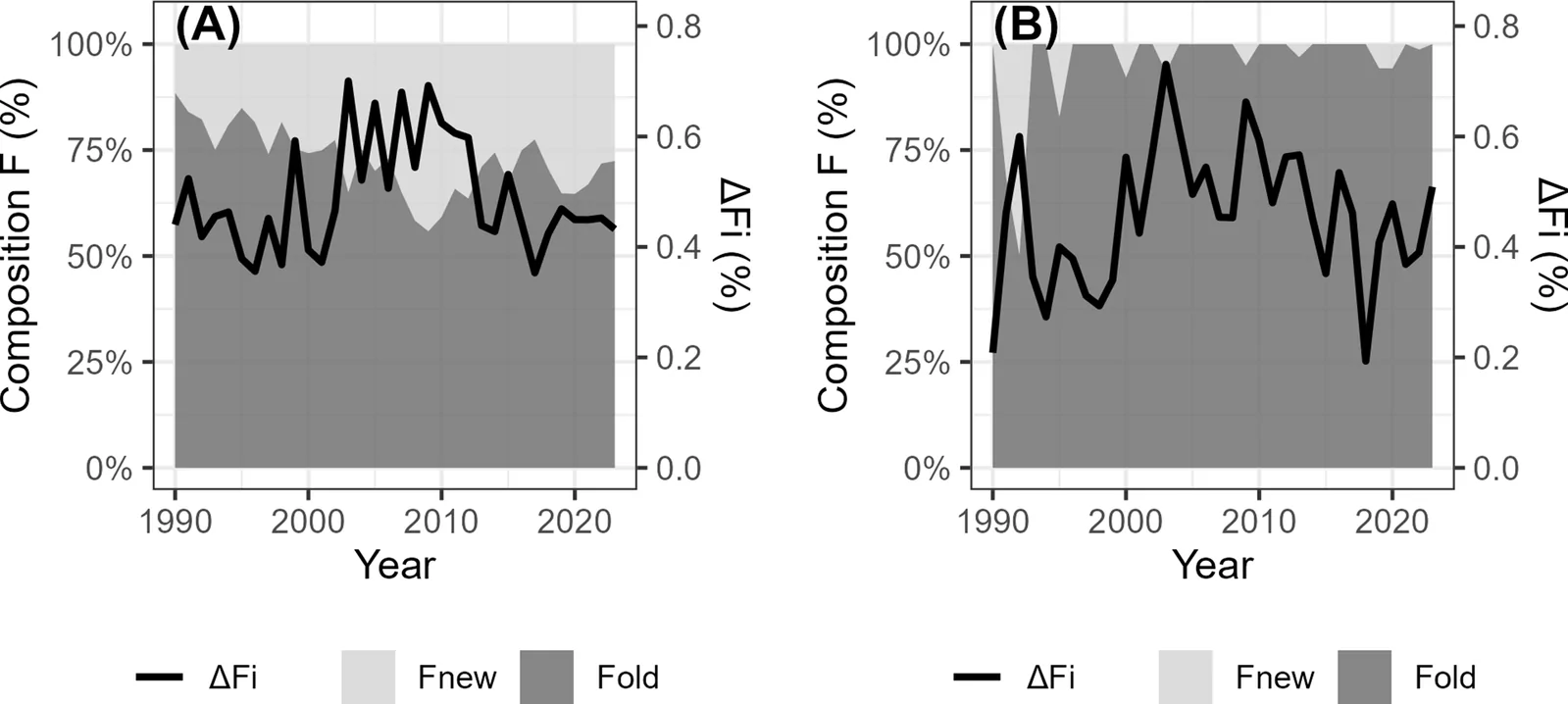
Discrimination of the inbreeding coefficient ($\:F\%$) into old ($\:{F}_{\mathrm{o}\mathrm{l}\mathrm{d}}$) and recent ($\:{F}_{\mathrm{n}\mathrm{e}\mathrm{w}}$) inbreeding, expressed as percentage of total inbreeding, and the average individual rate of inbreeding ($\:\varDelta\:Fi$) by birth year from 1990 to 2023 for the reference populations of the Caracu breed: (**A**) general population and (**B**) subpopulation

Figure 2 shows the composition of total inbreeding in each group, breaking down the genetic contributions of $\:{F}_{old}$ and $\:{F}_{\mathrm{n}\mathrm{e}\mathrm{w}}$ to * F*, as well as the rate of increase in $\:\varDelta\:Fi$. In the subpopulation, $\:\varDelta\:Fi\:$increased from 0.21% in 1990 to 0.51% in 2023. Inbreeding was mainly composed of $\:{F}_{\mathrm{o}\mathrm{l}\mathrm{d}}$ contributions, with a low contribution of $\:{F}_{\mathrm{n}\mathrm{e}\mathrm{w}}$, reflecting higher levels of $\:{F}_{\mathrm{o}\mathrm{l}\mathrm{d}}$ over the years. In the general population, $\:\varDelta\:Fi$ remained stable, ranging from 0.35% to 0.70%, with a value of 0.43% in 2023. There was a change in the composition of inbreeding over time, characterized by an increase in the proportion of $\:{F}_{\mathrm{n}\mathrm{e}\mathrm{w}}$, which ranged from 11.52% to 27.59% between 1990 and 2023. In contrast, the proportion of $\:{F}_{\mathrm{o}\mathrm{l}\mathrm{d}}$ decreased from 88.48% to 72.41% over the same period.

In the general population, the average * GI* was 6.2 years. No difference in * GI* was observed between parental groups, whether sire/dam to son or to daughter, with * GI* ranging from 6.2 to 6.5. In the subpopulation, the average * GI* was shorter (5 years) compared to the general population. In this subpopulation, there was a difference between paternal and maternal * GI*, with sires contributing to a shorter * GI*. The * GI* of sire-to-son and sire-to-daughter groups was similar (4.15 and 4.12, respectively). On the other hand, the highest dam-to-son * GI* was found in the subpopulation (6.66), which even exceeded the value observed in the general population (6.45). The dam-to-daughter * GI* (6.21) was shorter and closer to the mean value of the subpopulation. The * GI* metrics are shown in Table 3.

**Table 3:** Average generation interval and number of observations obtained for the different parental groups of the reference populations (general and subpopulation) of the Caracu breed

Group	*GI*_general population_	N_p_	*GI*_subpopulation_	N_s_
Mean	6.20	50,802	5.05	1,974
Sire	6.16	30,763	3.95	987
Dam	6.25	29,039	6.14	987
Sire-to-son	6.20	2,096	4.15	122
Sire-to-daughter	6.19	14,143	4.12	455
Dam-to-son	6.45	1,798	6.66	127
Dam-to-daughter	6.21	12,840	6.21	450

* GI* = generation interval; N_p_ = number of observations for the general population; N_s_ = number of observations for the subpopulation

Regarding $\:{f}_{e}$, $\:{f}_{a}$, and $\:{f}_{g}$, these measures increased in the general population between 1990 and 2023 from 45.3 to 133.3, from 35.23 to 93.67, and from 16.06 to 38.58, respectively. The highest values were observed in 2019, with 146.1 for $\:{f}_{e}$, 105.94 for $\:{f}_{a}$, and 43.24 for $\:{f}_{g}$. Consequently, * 1-GD* showed a continuous decline, decreasing from 3.11% in 1990 to 1.29% in 2023.

**Fig. 3:**
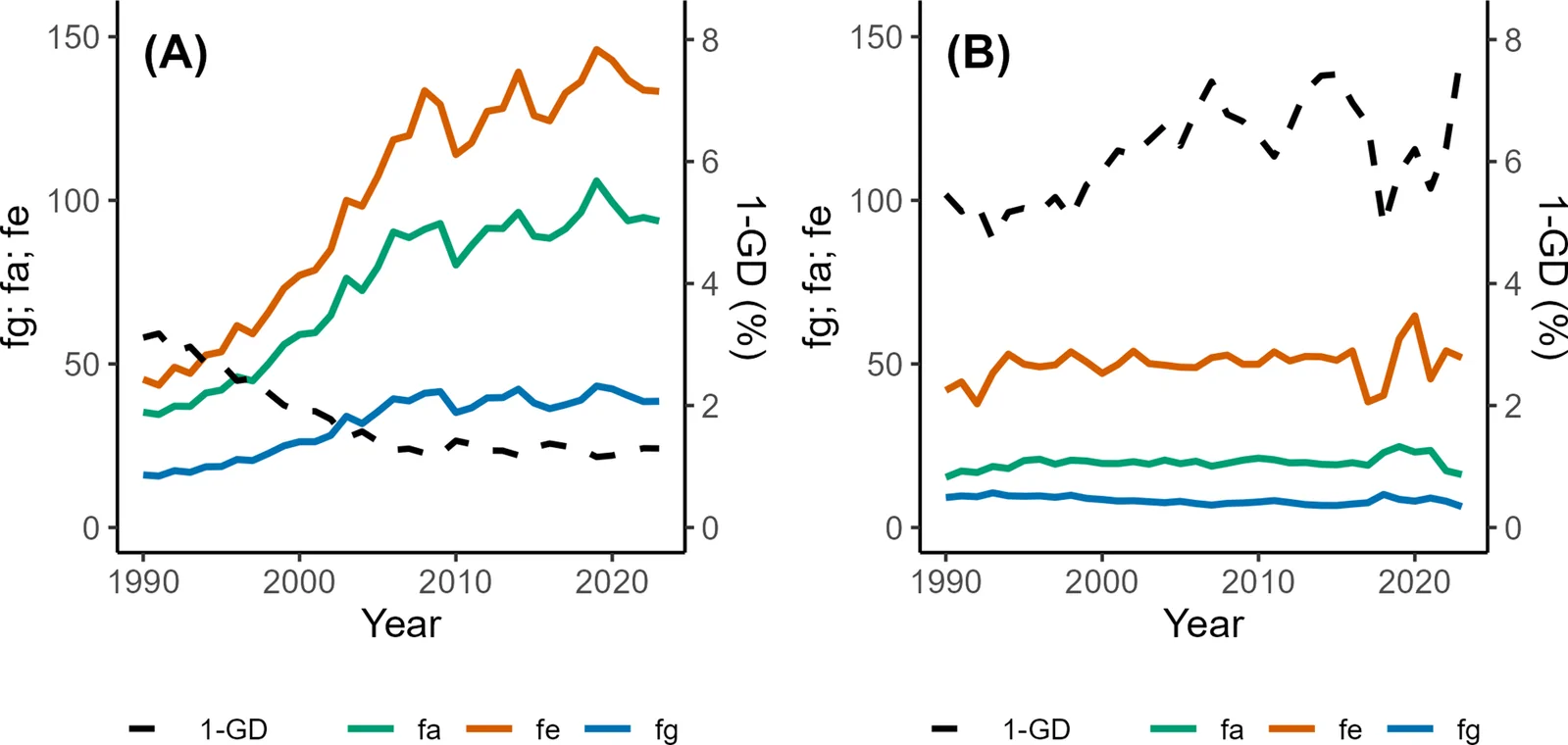
Effective number of founder genomes ($\:{f}_{g}$), effective number of ancestors ($\:{f}_{a}$), and effective number of founders ($\:{f}_{e}$), as well as loss of genetic diversity since the founder population ($\:1\:-\:GD$), in the reference populations of the Caracu breed evaluated from 1990 to 2023: (**A**) general population and (**B**) subpopulation

Considering the subpopulation, $\:{f}_{e}$ increased over the years, ranging from 41.9 in 1990 to 38.4 in 2017 and reaching a peak of 64.7 in 2020. Nevertheless, the final value in 2023 was 51.9, indicating high variation. Similarly, $\:{f}_{a}$ increased over time. Starting at 15.35, it reached a peak of 24.74 in 2019 before declining to 16.19 in 2023. There was a slight downward trend in $\:{f}_{g}$. Starting at 9.16, this measure peaked at 10.59 in 1993 and then gradually declined to 6.38 in 2023. On the other hand, there was a significant increase in * 1-GD*, starting at 5.46% in 1990 and exhibiting a fluctuating upward trend over the years, reaching its peak of 7.84% in 2023. These trends are illustrated in Fig. 3.

The majority of the 46 animals that accounted for 50% of the genetic diversity in the general population were sires (73.91%) (Supplementary Table 1). It is noteworthy that most of these animals belong to older generations. Only one animal, born in 2016, made a significant contribution. Although young, this animal showed a high connectivity with other animals.

Supplementary Table 2 lists the animals that contributed at least 50% of the genetic diversity to the subpopulation. The number was approximately four times lower than that of the general population. Ten of the 11 animals were males (90.91%). The group of the most representative ancestors included animals born between 1978 and 2014.

**Fig. 4:**
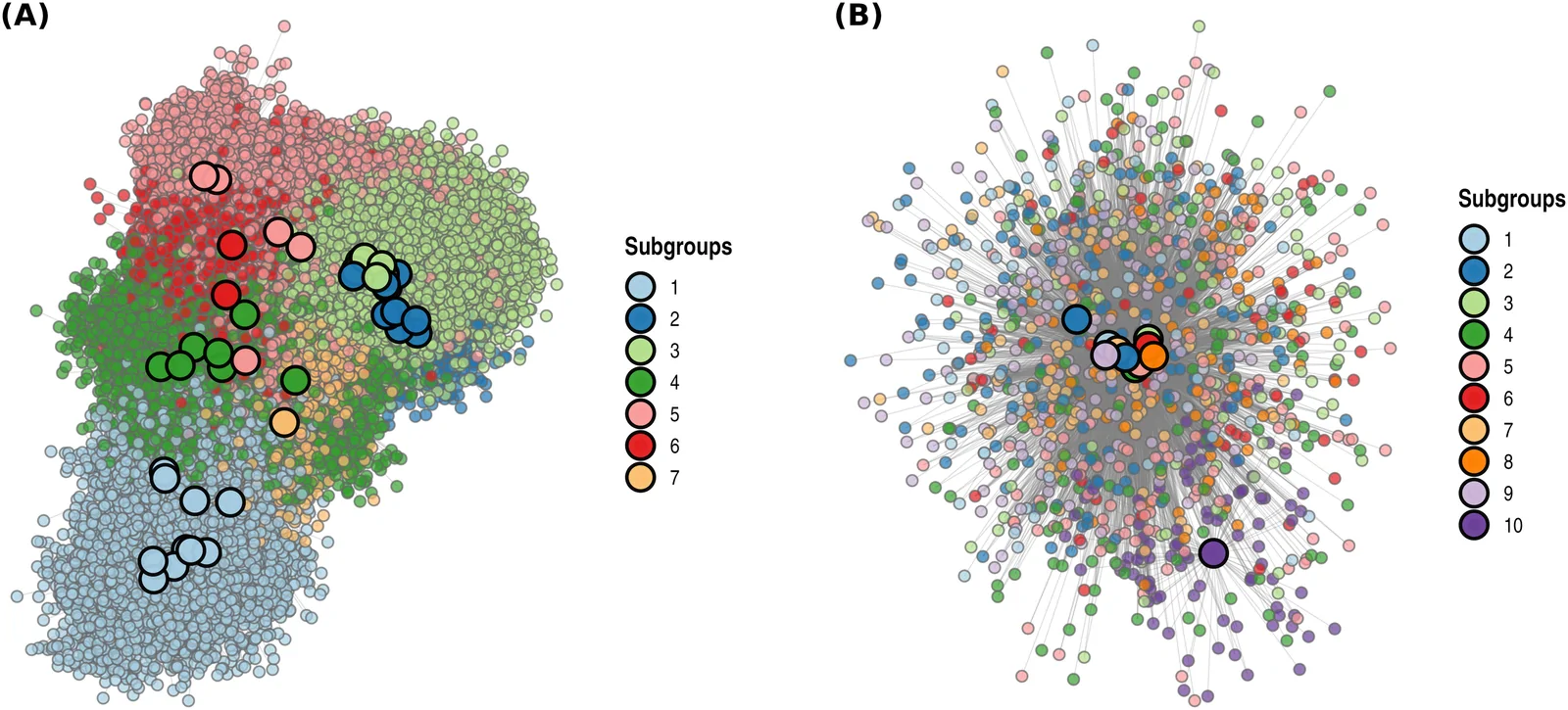
Kinship network of the reference populations of the Caracu breed: (**A**) general population and (**B**) subpopulation. Larger nodes represent animals that account for 50% of the genetic diversity, while smaller nodes represent the remaining individuals connected to them. Each grey edge indicates one pedigree relationship

The kinship network of the general population was characterized by a pattern of clustering, with individuals grouping into specific families identified by the detected subgroups (Fig. 4). Among the key ancestors, strong centrality was observed in certain clusters, for example group 3 (light green), in which individuals 25,534 (15,768 connections), 27,071 (12,843 connections), 31,802 (12,152 connections), and 24,582 (10,592 connections) exhibited the largest numbers of connections. These individuals were connected to almost 50% of the population, indicating broad overall influence. Other groups also contained ancestors that were relevant to the population structure, for example, group 4 (dark green) with individual 28,610 (2,884 interactions) and group 5 (pink), in which individuals 31,057 (6,492 interactions) and 25,749 (8,784 interactions) were highly connected. These patterns are consistent with the presence of structured clusters and the role of central ancestors in maintaining the network’s connectivity and organization.

The kinship network of the subpopulation exhibited less distinct clustering compared to the general population, except for one distant cluster. Individuals 272 (877 connections), 1025 (860 connections), and 1084 (835 connections) were the most connected ancestors. These individuals were positioned at the center of the network and were a key connection among nodes between different families. In contrast, individual 199 (purple), with only 90 interactions, exhibited a low level of connectivity and was located farther from the central area of the graph.

## Discussion

Multiple metrics were evaluated to describe the population structure and inbreeding in a general Caracu cattle population. The same parameters were used for a closed subpopulation of these animals, which has been selected for body weight for over 40 years. Although comparing the general population with a subpopulation allows us to identify differences in inbreeding and genetic diversity patterns, it is important to consider structural differences between these populations such as population size, pedigree depth, historical period during which records were obtained, selection objectives, and the strategy used for introducing founders. These characteristics can directly influence the estimated pedigree parameters. Thus, some of the observed differences may reflect not only the effect of the breeding strategy but also demographic and structural factors associated with population size and the degree of population closure.

The subpopulation exhibited higher * ECG* values, which are consistent with those reported for well-documented or well-managed populations such as local French breeds (Leroy et al. 2013) and Brazilian Zebu cattle (Santana et al. 2016), as well as a closed Nellore herd with high pedigree completeness (Oliveira et al. 2011). In contrast, the general population showed lower * ECG* values, although these values still exceeded those reported for some local Colombian breeds (Martínez et al. 2008). These differences in * ECG* reflect variations in pedigree depth and completeness across populations and are closely related to their structure and management. Higher * ECG* values, as observed in the subpopulation and in closed or more intensively managed herds, indicate more complete pedigree information, improving the reliability of inbreeding estimates and genetic analyses. In contrast, the lower * ECG* in the general population probably reflect its larger size, longer time span, and the integration of multiple herds with heterogeneous recording practices, which can limit pedigree depth. Therefore, these differences are not only biological but also indicate variations in data recording and breeding management.

The increase in the number of births in the general population between 1970s and 2000s indicates a period of expansion associated with greater acceptance of the breed, the consolidation of breeding programs, and the possible geographic dispersion of herds. Such growth phases are typically linked to improved organization of recording systems and increased participation of breeders, which can increase population size and the availability of pedigree information. However, the decline in the number of births, especially after 2000s, warrants attention since it may have direct implications for genetic diversity, effective population size, and inbreeding levels. A sustained decrease in registrations can intensify genetic drift and increase the probability of mating among related individuals, particularly if the number of active sires is reduced.

Similar patterns have been reported for other local breeds. Carolino et al. (2020) described an increase in the number of registered calves of the local Portuguese Mertolenga breed from the 1990s to 2007, followed by a progressive decline through 2019, suggesting that initial expansion phases may not be sustained over time. In contrast, Santana et al. (2016) reported an increase in the number of animals of the Nellore, Guzerá, and Brahman breeds in Brazil between 2006 and 2012, indicating that population trajectories can differ substantially depending on breed characteristics and market demand. This divergence may reflect shifts in producer preferences, as highlighted by McManus et al. (2010) who reported a trend toward the adoption of Zebu breeds in Brazil as a result of their adaptation and productive performance under tropical conditions. Tavares and Almeida (2024) found a decline in the Portuguese Mertolenga breed to be associated with the introduction of non-indigenous breeds such as Limousin and Charolais, reinforcing the role of breed substitution as a driver of population contraction.

Overall, these results suggest that demographic trends in the Caracu population are likely influenced not only by biological factors but also by economic and management decisions. Changes in breed preference, the introduction of alternative genetic resources, and fluctuations in breeding program intensity may all contribute to the observed decline in births. These factors should be considered when evaluating long-term genetic diversity and designing conservation or breeding strategies.

The * F* value was similar between the general population and subpopulation. Although these values are higher than those reported by Martinez et al. (2008) for native Colombian breeds, it is important to note that * F* remained relatively low, even for a small population, and was lower than the value found for the Romosinuano breed (Martinez et al., 2008). Pereira et al. (2005) evaluated the same subpopulation as used in the present study up to 2002 and obtained an * F* of 1.95%. Thus, over 10 years of selection, * F* increased by only 0.4% within the subpopulation, a finding that suggests controlled inbreeding levels over time and likely reflects the applied mating management.

Both populations evaluated in the present study are subject to independent genetic evaluation programs based on the BLUP (Henderson 1975; Aguilar et al. 2010; Christensen and Lund 2010). Although genetic evaluation and the subsequent use of breeding values for selection can contribute to increased rates of inbreeding when genetic relationships among candidates are not considered in mating decisions, the IZ subpopulation adopts a mating decisions, the IZ subpopulation adopts a mating strategy aimed at minimizing inbreeding and controlling the accumulation of relatedness among breeding animals (Ligori et al. 2025; Bem et al. 2024). Therefore, the lower F observed in the subpopulation compared with the general population may be associated with the controlled mating management adopted in the IZ subpopulation. In contrast, the general population does not follow a mating strategy aimed specifically at minimizing inbreeding, which may contribute to the higher accumulation of inbreeding observed in this population.

Although the subpopulation exhibited a lower * F* than the general population, this finding does not necessarily indicate a more favorable genetic condition. The * F* value reflects accumulated inbreeding and does not capture the rate at which inbreeding is increasing or the associated future risk, which are better described by $\:{\varDelta\:F}_{i}$ and * NeFi*. In small and closed populations, these metrics provide a more sensitive assessment of genetic dynamics because they account for recent changes in relatedness and the effective number of breeding individuals, enabling the detection of ongoing increases in inbreeding that may not yet be reflected in * F*. The maximum * F* value was also higher in the general population (44.34%), a result similar to that found for the Brazilian Sindi breed (Santana et al. 2016), which also has a small population size. Similar maximum * F* values were reported for the Nellore *mocho* breed, which has historically faced challenges because of its small number of animals (Faria et al. 2002).

In the Caracu breed, the results suggest the occurrence of higher inbreeding levels in specific herds within the general population, which may represent structured subgroups within the population that correspond to lineages focused on milk (El Faro and Albuquerque 2003; Campos et al. 2017) and meat production (Bonilha et al. 2012). The use of a limited number of sires, especially in dairy herds, may have contributed to this pattern, as also reported by Santana et al. (2014) for the Gir breed.

The higher * C* value observed in the subpopulation indicates a greater probability of mating between related individuals, even if the animals were kept under conditions of random mating. This result was expected since the subpopulation corresponds to a closed herd, in which the introduction of animals is limited, a fact that progressively reduces the chances of mating between unrelated individuals with each generation. In addition, the combination of a closed population structure and genetic selection (BLUP) may increase the concentration of genetic contributions from a limited number of related animals. When seletion favors individuals with high estimated breeding values, the repeated use of these animals and their descendants may increase C (Woolliams et al. 2015), particularly in closed population (Bem at al., 2024). On the other hand, the general population had lower * C* and higher * F* values than the subpopulation. This finding may be related to the recurrent use of certain sires within specific family groups (Fig. 4). Such pattern suggests that some animals and their offspring were used more intensively in some herds, promoting an increase in * F* even in a population with a lower average * C* level. This scenario may be related to attempts to fix phenotypic traits and the absence of mating control strategies aimed at reducing inbreeding in some herds. Carolino et al. (2020) reported a $\:C\:$of 2.05% for the indigenous Portuguese Mertolenga breed evaluated between 2015 and 2019, a value higher than that estimated for the general Caracu breed population in the present study. This result suggests a lower degree of kinship among individuals of the Caracu breed compared to other native breeds, which is an important aspect for the preservation of genetic diversity.

In 2018, there was a decrease in * C* in the subpopulation, followed by a slight decrease in * F*, which led to an increase in the *α* value. This pattern coincides with the period when individuals were introduced into the subpopulation for promoting the polled gene. Despite the introduction of animals into the population, which promoted a reduction in$\:\:C$, the results indicate the absence of a proportional decrease in * F*. This finding may reflect the small size and genetic structure of the Caracu population in Brazil, in which newly introduced individuals may still share common ancestry with the existing population.

The $\:{F}_{new}$ value for the general population corroborates previous results and indicates a greater contribution of recent inbreeding, suggesting that inbred matings dated back up to three generations. In contrast, the contribution of $\:{F}_{new}$ was low in the subpopulation, with a higher proportion of inbreeding being explained by an ancient common ancestor within the population. Similar results have been reported by Verardo et al. (2021), who analyzed runs of homozygosity to characterize inbreeding in the Caracu breed using individual animals from different herds. The authors found that more than 50% of the runs were smaller than 5 MB, characterizing historical inbreeding associated with more distant ancestors. This finding suggests that pedigree-based inbreeding in the subpopulation is consistent with patterns of historical genome-based inbreeding.

The annual values of $\:\alpha\:$ showed slightly different patterns between the two populations. In the general population, the results revealed fluctuations with initial negative values, indicating deviations from random mating that possibly reflect mating strategies aimed at avoiding inbreeding. However, the *α* value was positive in 2003, negative in 2004, and again positive in 2007. Despite this fluctuation, the values remained close to zero. In the subpopulation, the *α* values fluctuated negatively, indicating deviation from random mating toward avoidance of inbreeding. This result suggests that matings are directed to avoid inbreeding in the subpopulation since, even with the increase in * C*, * F* did not follow the same upward trend. Santana et al. (2016) reported fluctuations in *α* for the Brahman, Gir, Guzerá, Indubrasil, Nellore, Sindi, and Tabapuã Zebu breeds; however, the values were positive. Stachowicz et al. (2011) obtained positive results for Jersey and Canadian Holstein animals, with a slight downward trend after 1988. Our findings support the interpretation that breeding practices adopted by Caracu breeders and breeding programs have been effective in maintaining appropriate mating strategies to limit inbreeding when compared to other breeds.

The mean $\:{\varDelta\:F}_{i}$ was similar in the two populations, although significant fluctuations occurred over the years. Gonzales et al. (2022) found lower values for the Braford breed, along with lower levels of other inbreeding parameters. In that study, the total evaluated population was 358,041 and the reference population contained 115,757 animals, providing more opportunities for non-inbred matings compared to the Caracu breed analyzed. The latter has a smaller number of animals and a higher * C* value (general Caracu breed population: 1.2% vs. Braford breed population: 0.002%).

The * NeFi* was similar in the two populations, with values below 100. According to the FAO (2021), such values indicate an increased risk of genetic diversity loss within a population. The low * NeFi* in the populations evaluated may be explained by the historical formation of the Caracu breed (McManus et al. 2010; Verardo et al. 2021). The number of founders was higher in the general population, as expected given that the subpopulation is nested within it. Similar * NeFi* values have been reported for the Devon breed, which had a population size twice that of the general Caracu breed (Piccoli et al. 2014). However, in the Devon breed, the founder individuals accounted for 3.22% of the population, while this value was 8.70% in the general Caracu breed. Thus, the Caracu breed had a higher proportion of founders than other breeds described in the literature.

The * GI* observed in the two populations suggest relatively rapid generational turnover, particularly when compared with intervals reported for other cattle populations such as the Mertolenga breed and Brazilian Zebu breeds (Santana et al. 2016; Carolino et al. 2020), which are generally longer. Shorter * GI* are typically associated with more dynamic replacement strategies and can contribute to faster genetic progress. In contrast, increases in * GI* are often associated with the prolonged use of older proven sires, especially through artificial insemination. The use of semen from elite or historically influential sires can extend the average parental age at reproduction, as reported for the Gir breed, in which the intensive use of older sires resulted in longer * GI* (Santana et al. 2014). Differences in * GI* between males and females are also influenced by herd management practices. A higher turnover of breeding males tends to reduce the male * GI*, whereas longer retention of females contributes to longer * GI*. Additionally, factors such as the use of a limited number of sires and semen from deceased animals, as well as extended reproductive lifespans of females, have been shown to increase the overall * GI* (Santana et al. 2014; González et al. 2022).

The ratios of $\:{f}_{e}$, $\:{f}_{a}$ and $\:{f}_{g}$, which correspond to the effective number of founders, effective number of ancestors, and effective number of founder genomes, also differed among populations. In the general population, the number of founders, ancestors, and founder genomes tended to increase, demonstrating changes in the distribution of genetic contributions over time. This finding may reflect changes in pedigree recording and population structure, possibly associated with the growing adoption of breeding programs, which increases the number of animals registered in the pedigree. The values of these parameters thus remained constant in the subpopulation, possibly because of the implementation of targeted matings to prevent the loss of genetic diversity.

The mean $\:{f}_{e}$ values for the general population were lower than those reported by Piccoli et al. (2014) for British taurine breeds (Angus, Hereford, and Shorthorn), except for the Devon breed, which showed a lower mean. For the Gir breed, Santana et al. (2014) reported an $\:{f}_{e}$ value similar to that observed for the general Caracu population. The Gir breed shares a similar limitation with the Caracu breed, with both breeds originating from a small number of founder individuals, which is a matter of concern for the management of genetic diversity (McManus et al. 2010). Compared to the Caracu subpopulation, all breeds described by Piccoli et al. (2014) and Santana et al. (2014) had higher $\:{f}_{e}$ values, which was expected given that the subpopulation is a closed herd comprising a small portion of the general population.

For $\:{f}_{a}$, the general population showed higher values than those reported for other breeds such as Angus, Devon, Hereford, Shorthorn (Piccoli et al. 2014), Gir (Santana et al. 2014, 2016), Brahman, Guzerá, Indubrasil, Nellore, Sindi, Tabapuã (Santana et al. 2016), Bonsmara, and Marchigiana (Santana et al. 2012). This finding indicates that, although the population studied had a smaller effective number of founders than the other breeds, there was less loss of founder contributions across generations. The reduction in $\:{f}_{a}$ relative to $\:{f}_{e}$ and the decrease in $\:{f}_{g}$ indicate bottleneck effects and the influence of genetic drift over time. These results suggest that, although bottleneck effects are present in the general population, their impact may have been less pronounced compared to the other breeds. However, it is important to note that all of the aforementioned breeds are more widely distributed across diverse production systems, a fact that inherently promotes a larger number of founders and greater initial genetic diversity.

Measures related to genetic diversity loss, such as * 1-GD*, $\:1-{GD}^{*}$, and $\:{GD}^{*}-GD$, were more pronounced in the subpopulation. This is commonly observed in closed populations with restricted gene flow, particularly in those under genetic selection (FAO 2021). The reduction in genetic diversity loss observed in the general population over the years may be related to the recent establishment of genetic evaluations for this population. Despite the increasing use of genetic selection by producers, many of them do not apply breeding values to guide sire selection and many herds do not participate in breeding programs of the breed. However, the gradual adoption of these programs by new producers has increased the number of animals included in the pedigree, promoting better connectivity among herds, which may have contributed to the changes in genetic diversity patterns observed in more recent generations. Thus, analyses that include genomic information, for example the study conducted by Verardo et al. (2021) that used a larger number of animals from different herds, may provide complementary insights because genomic data permit to account for Mendelian segregation and the realized relationship, even without pedigree information (Van Raden, 2008).

The organization of individuals according to their degree of relatedness provided results consistent with those obtained in the inbreeding analyses. These results showed that, in each population, some animals were intensively used within specific groups, without considering the associated loss of genetic diversity. This fact helps explain why the general population exhibits a higher inbreeding coefficient, even at lower average relatedness. Similar results have been reported by Verardo et al. (2021), who analyzed genotypes from a limited number of animals of the Caracu breed selected for meat production and another group selected for milk production. Principal component analysis performed by the authors revealed that different selection directions resulted in distinct clusters of animals. Similarly, Reverter (2019) analyzed pedigree data from Brahman, tropical composite, Angus, and Nellore breeds and found different network structures that varied according to animal location, a finding possibly linked to different selection objectives. In the present study, the distribution of animals was more homogeneous in the subpopulation, with the most influential individuals displaying centrality measures that reflect the structure of the entire population. A single individual exhibited a markedly distinct centrality since it was introduced from another farm to incorporate the polled gene into the subpopulation. This individual lacked pedigree information, a fact that prevented it from forming pedigree connections with animals present before its introduction. Even without these prior connections, this individual contributed to the genetic diversity of the group through its descendants.

Taken together, these results have direct implications for breeding and conservation strategies in the Caracu breed. The contrasting patterns observed between the general population and the closed subpopulation indicate that maintaining genetic diversity requires different context-specific approaches. In the general population, efforts should prioritize the more balanced use of sires across herds and the avoidance of excessive concentration of genetic contributions, thereby reducing the risk of high inbreeding peaks. In the subpopulation, in which genetic drift and coancestry are more pronounced, the implementation of controlled mating schemes, the periodic introduction of unrelated or low-related individuals, and the integration of genomic information are essential to mitigate diversity loss. Moreover, the identification of key ancestors and network structure provides a practical framework to guide mating decisions by minimizing relatedness while preserving valuable lineages. Overall, the application of these strategies can enhance the long-term sustainability of breeding programs, ensuring that genetic gains are achieved without compromising the adaptive potential and resilience of the breed.

## Conclusion

Pedigree analysis revealed contrasting patterns of genetic structure between the general Caracu population and the closed subpopulation. Although mean inbreeding levels were similar between populations, the general population presented higher maximum inbreeding, whereas the subpopulation exhibited higher average coancestry and greater losses of genetic diversity. These differences indicate that mean inbreeding alone is insufficient to characterize the genetic status of the populations. The greater coancestry and unequal ancestral contributions observed in the subpopulation may be associated with its closed structure and long-term selection for body weight; such factors may favor the use of genetically related individuals and, consequently, increase genetic drift and reduce the effective contribution of founders across generations.

The contrasting patterns observed between the populations highlight the importance of considering multiple pedigree indicators when monitoring genetic diversity in Caracu cattle. In the general population, the presence of highly connected individuals and distinct family clusters suggests that the concentration of genetic contributions from influential sires should be monitored to avoid excessive relatedness. In the closed subpopulation, greater attention should be given to the accumulation of coancestry and the loss of genetic diversity resulting from genetic drift and restricted ancestral contributions. Therefore, population-specific mating strategies that promote more balanced genetic contributions while maintaining the desired response to selection may help preserve genetic diversity and limit the accumulation of inbreeding in future generations.

## Supplementary Information

Below is the link to the electronic supplementary material.

Supplementary Material 1

## Data Availability

The datasets generated and analyzed during the current study are not publicly available but may be available from the corresponding author upon reasonable request and institutional approval.
