# Supplementary material for: Genetic diversity of Caracu cattle reveals contrasting trends between general and closed populations
Source: Trop Anim Health Prod. 2026 Oct 8;58(8):584. doi: 10.1007/s11250-026-05351-9 (PMC13649896; doi:10.1007/s11250-026-05351-9)
Supplement: Supplementary file 1 [file 11250_2026_5351_MOESM1_ESM.docx]

**Supplementary Table 1.** Animals accounting for 50% of the genetic diversity in the reference general population of the evaluated Caracu breed.

| **Rank** | **ID** | **Sex** | **Birth year** | **Marginal contribution (%)** | **Cumulative contribution (%)** |
| --- | --- | --- | --- | --- | --- |
| 1 | 25534 | male | 1951 | 5,68 | 5,68 |
| 2 | 31802 | male | 1957 | 2,81 | 8,49 |
| 3 | 32476 | male | 1996 | 2,10 | 10,59 |
| 4 | 7279 | male | 2008 | 2,07 | 12,67 |
| 5 | 25749 | female | 1960 | 2,01 | 14,68 |
| 6 | 26494 | male | 1965 | 1,65 | 16,33 |
| 7 | 11249 | male | 2012 | 1,64 | 17,96 |
| 8 | 28610 | male | 1978 | 1,53 | 19,50 |
| 9 | 1546 | male | 2001 | 1,51 | 21,00 |
| 10 | 7121 | male | 2006 | 1,42 | 22,43 |
| 11 | 25666 | female | 1950 | 1,39 | 23,82 |
| 12 | 2165 | male | 2002 | 1,32 | 25,14 |
| 13 | 27136 | male | 1982 | 1,31 | 26,45 |
| 14 | 25615 | female | 1956 | 1,27 | 27,72 |
| 15 | 25509 | male | 1940 | 1,25 | 28,97 |
| 16 | 24582 | female | 1937 | 1,20 | 30,17 |
| 17 | 28662 | male | 1985 | 1,12 | 31,29 |
| 18 | 33187 | female | 1998 | 1,02 | 32,31 |
| 19 | 25519 | male | 1940 | 0,99 | 33,30 |
| 20 | 3017 | male | 2003 | 0,90 | 34,20 |
| 21 | 26053 | female | 1961 | 0,89 | 35,10 |
| 22 | 27826 | male | 1985 | 0,88 | 35,98 |
| 23 | 5038 | male | 2005 | 0,88 | 36,87 |
| 24 | 5538 | male | 2003 | 0,80 | 37,66 |
| 25 | 31057 | male | 1994 | 0,76 | 38,42 |
| 26 | 32423 | male | 2016 | 0,75 | 39,17 |
| 27 | 25514 | male | 1945 | 0,72 | 39,89 |
| 28 | 8544 | male | 2009 | 0,71 | 40,60 |
| 29 | 29109 | male | 1985 | 0,68 | 41,27 |
| 30 | 3786 | male | 2004 | 0,65 | 41,92 |
| 31 | 26464 | male | 1961 | 0,61 | 42,53 |
| 32 | 7148 | female | 2008 | 0,60 | 43,13 |
| 33 | 212 | male | 1998 | 0,59 | 43,72 |
| 34 | 29070 | male | 1990 | 0,57 | 44,29 |
| 35 | 6665 | male | 2007 | 0,56 | 44,84 |
| 36 | 33389 | female | 1997 | 0,55 | 45,40 |
| 37 | 25885 | female | 1955 | 0,55 | 45,95 |
| 38 | 2488 | male | 1999 | 0,54 | 46,49 |
| 39 | 31664 | male | 1955 | 0,54 | 47,03 |
| 40 | 3454 | male | 2003 | 0,53 | 47,56 |
| 41 | 25614 | female | 1950 | 0,52 | 48,09 |
| 42 | 26496 | male | 1965 | 0,51 | 48,60 |
| 43 | 27071 | male | 1978 | 0,51 | 49,11 |
| 44 | 25555 | female | 1943 | 0,51 | 49,61 |
| 45 | 6919 | male | 2006 | 0,50 | 50,12 |
| 46 | 1877 | female | 2002 | 0,49 | 50,61 |

ID= renumbered animal.

**Supplementary Table 2.** Animals accounting for 50% of the genetic diversity in the reference subpopulation of the evaluated Caracu breed.

| **Rank** | **ID** | **Sex** | **Birth year** | **Marginal contribution (%)** | **Cumulative contribution (%)** |
| --- | --- | --- | --- | --- | --- |
| 1 | 1007 | male | 1985 | 6,54 | 6,54 |
| 2 | 1084 | male | 1991 | 6,12 | 12,66 |
| 3 | 903 | male | 1978 | 5,37 | 18,02 |
| 4 | 1025 | male | 1986 | 5,35 | 23,37 |
| 5 | 199 | male | 2011 | 5,25 | 28,62 |
| 6 | 937 | male | 1978 | 4,99 | 33,61 |
| 7 | 998 | female | 1983 | 4,71 | 38,32 |
| 8 | 871 | male | 1978 | 4,03 | 42,35 |
| 9 | 1023 | male | 1986 | 3,83 | 46,19 |
| 10 | 272 | male | 2014 | 3,48 | 49,67 |
| 11 | 161 | male | 2008 | 3,00 | 52,67 |

ID= renumbered animal.
